# Navigating Perioperative Anticoagulation Challenges in a Cancer Patient With Deep Vein Thrombosis: A Case Report

**DOI:** 10.7759/cureus.100732

**Published:** 2026-01-04

**Authors:** Jacob George Binoy, Greeshma George, Dhanraj Parambeth, Faiz Abdul Rahman

**Affiliations:** 1 Trauma and Orthopaedics, Cumberland Infirmary, Carlisle, GBR; 2 Orthogeriatrics, Cumberland Infirmary, Carlisle, GBR; 3 Geriatrics, Midland Metropolitan University Hospital, Birmingham, GBR

**Keywords:** anticoagulation, cancer-associated thrombosis, deep vein thrombosis, pathological fracture, perioperative anticoagulation

## Abstract

Deep vein thrombosis (DVT) in cancer patients presents unique challenges, especially in the perioperative period, where anticoagulation must be carefully balanced to minimize both thrombotic and bleeding risks. This case highlights the complexity of managing DVT in an elderly patient with metastatic urothelial carcinoma of the bladder in the setting of a pathological femur fracture. An 80-year-old woman with metastatic urothelial carcinoma of the bladder presented with a pathological left femur fracture after trivial trauma. Multimodal imaging confirmed the fracture and incidentally revealed a right common femoral vein DVT along with disease progression, including early metastatic spread to the neural canal. Given the need for urgent surgery, full anticoagulation posed a bleeding risk, while withholding it increased the risk of embolism. To balance this, an inferior vena cava (IVC) filter was placed, and enoxaparin was initiated with a personalized perioperative regimen. Surgical fixation was delayed due to unforeseen circumstances, requiring further anticoagulation modifications. Postoperatively, wound concerns and hemoglobin drop necessitated transfusions and anticoagulation adjustments. Anemia workup identified low folate as one among many contributing factors, prompting supplementation. The patient was stabilized and transitioned to lifelong apixaban before discharge. This case underscores the complexities of anticoagulation in oncologic orthopedic patients, highlighting the individualized approach and real-time adjustments in anticoagulation management to balance thrombotic and bleeding risks, especially in light of unforeseen clinical developments.

## Introduction

Venous thromboembolism (VTE), comprising deep vein thrombosis (DVT) and pulmonary embolism (PE), is a common and serious complication in cancer patients, with malignancy increasing the risk of VTE by approximately four to seven times compared to the general population [1]. This risk is further amplified during the perioperative period due to factors such as immobility, surgical stress, and the underlying hypercoagulable state in cancer [2]. Anticoagulation remains the mainstay of treatment and prophylaxis for VTE; however, in the perioperative setting, especially in oncology patients, clinicians must balance the need to prevent thromboembolism against the increased risk of bleeding [3]. According to the American Society of Clinical Oncology (ASCO) guidelines, pharmacological thromboprophylaxis with low molecular weight heparin (LMWH) or unfractionated heparin (UFH) is recommended for cancer patients undergoing major surgery unless contraindicated due to active bleeding or significant bleeding risk [3]. Extended prophylaxis for up to four weeks postoperatively is advised in high-risk patients, such as those with limited mobility, obesity, or prior VTE [4]. When anticoagulation is contraindicated, the use of inferior vena cava (IVC) filters is considered to prevent PE [5]. However, their use in oncology remains controversial. Some studies report increased PE-free survival with IVC filter placement in selected high-risk cancer patients [6], while others caution against their routine use due to possible complications and uncertain long-term benefit [7]. Most guidelines suggest IVC filters be reserved for cases where anticoagulation is absolutely contraindicated or ineffective [8]. This case report highlights the multifaceted challenges of anticoagulation management in an elderly oncologic patient with an incidental DVT, requiring urgent surgical fixation for a pathological femoral fracture.

## Case presentation

An 80-year-old woman with a medical history of metastatic urothelial carcinoma of the bladder, previously treated with chemotherapy (three out of five cycles) and radiotherapy (one session), presented with severe left thigh pain after sitting down, having heard a snap sound without any significant trauma. She had been independent until two months prior, using a Zimmer frame for mobility for the last month.

**Figure 1:**
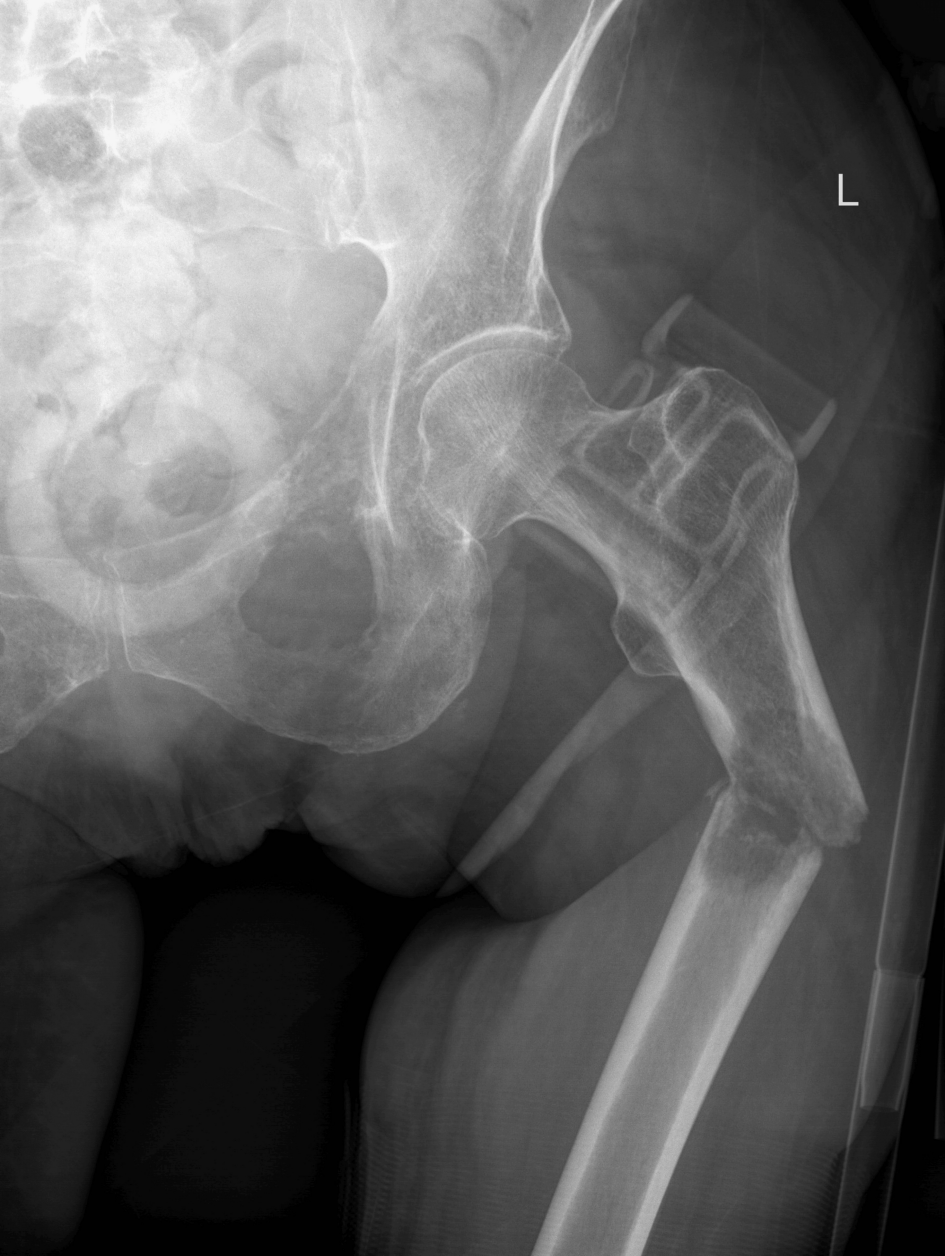
X-ray of the right hip, anteroposterior view, showing a left femoral shaft fracture

On admission, X-ray of the pelvis and left hip revealed a pathological fracture of the left femur, which occurred due to trivial trauma (Figure 1).

**Figure 2:**
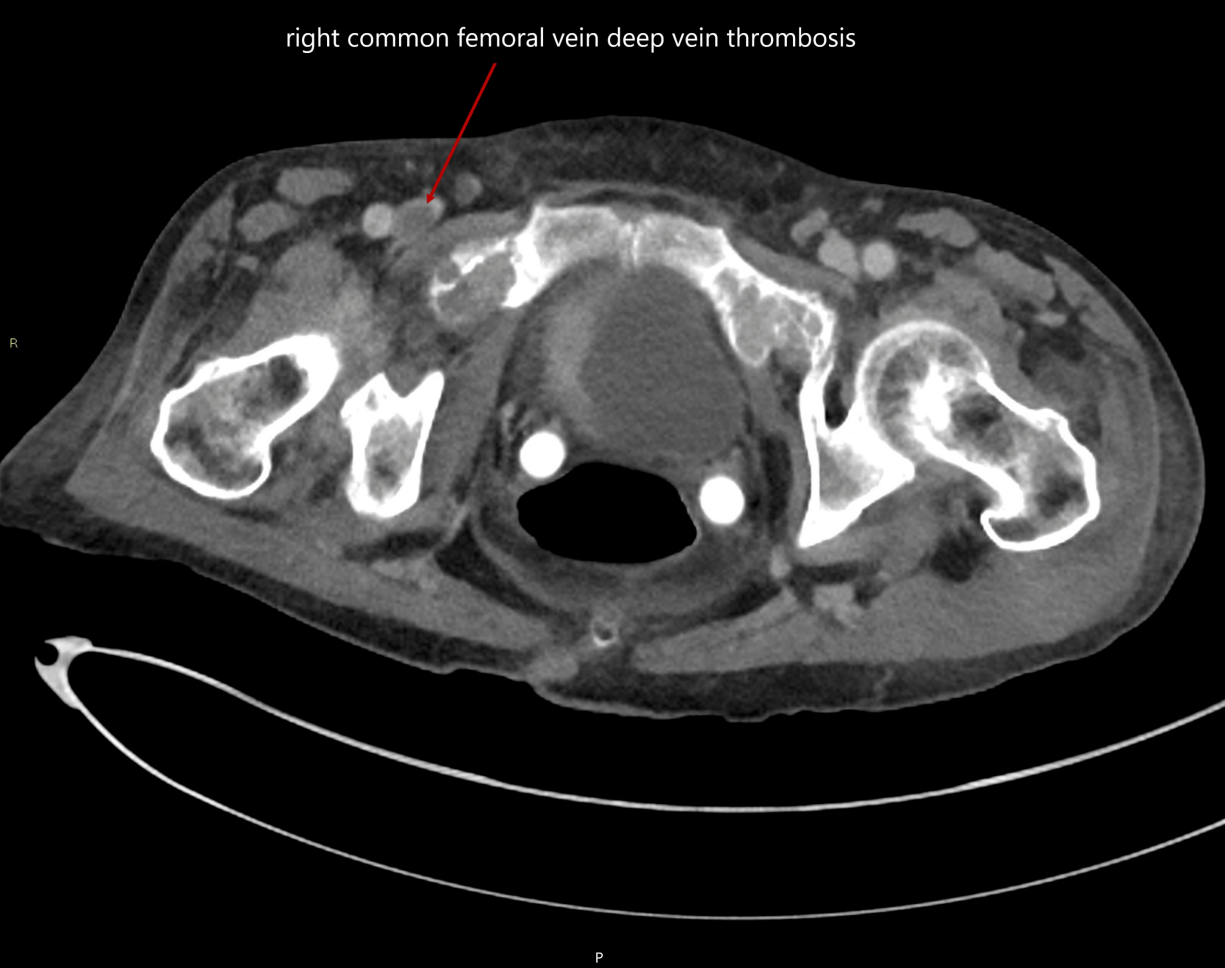
CT of the chest, abdomen, and pelvis with contrast, axial section, showing a right common femoral vein deep vein thrombosis CT: computed tomography

**Figure 3:**
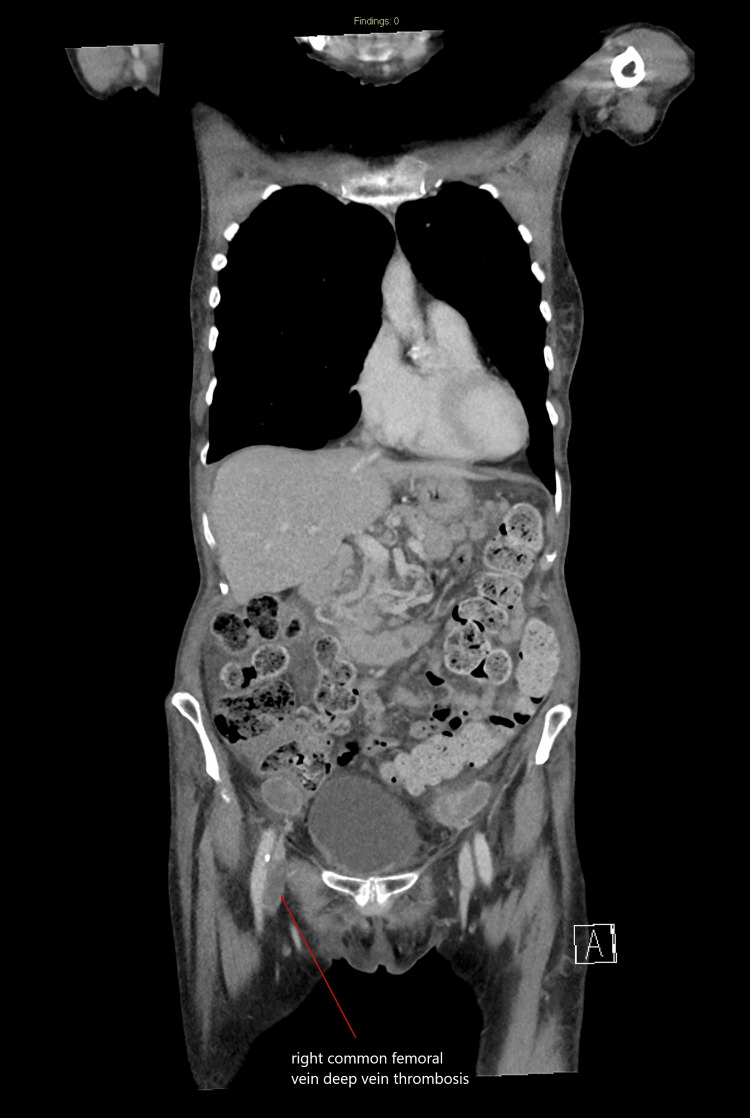
CT of the chest, abdomen, and pelvis with contrast, coronal section, showing a right common femoral vein deep vein thrombosis CT: computed tomography

A CT scan of the chest, abdomen, and pelvis was requested by the oncologist to assess disease progression and to evaluate any metastatic involvement. The CT scan revealed an incidental right common femoral vein DVT and early metastatic spread to the neural canal at the lumbar level (Figures 2-3).

On admission, the patient's hemoglobin was 76 g/L, prompting the transfusion of one unit of packed red blood cells (PRBC). A second unit was administered on hospital day 2 for pre-surgical optimization, resulting in an improved hemoglobin level of 104 g/L prior to surgery.

The hematology team was consulted to assess anticoagulation needs. A therapeutic dose of enoxaparin was initiated at 80 mg daily (1.5 mg/kg; patient weight 54 kg) following hematology input. The plan was to withhold anticoagulation 24 hours prior to surgery to minimize bleeding risk. Postoperatively, prophylactic enoxaparin was to be restarted six hours after surgery, with full therapeutic anticoagulation resumed from postoperative day 1. This involved enoxaparin 40 mg twice daily on postoperative days 1 and 2, followed by a switch to 80 mg once daily in the evening from postoperative day 3 onward. As anticoagulation had to be withheld around the time of surgery, an IVC filter was inserted on hospital day 2 by interventional radiology to prevent PE, and it was advised to remain in place permanently.

**Figure 4:**
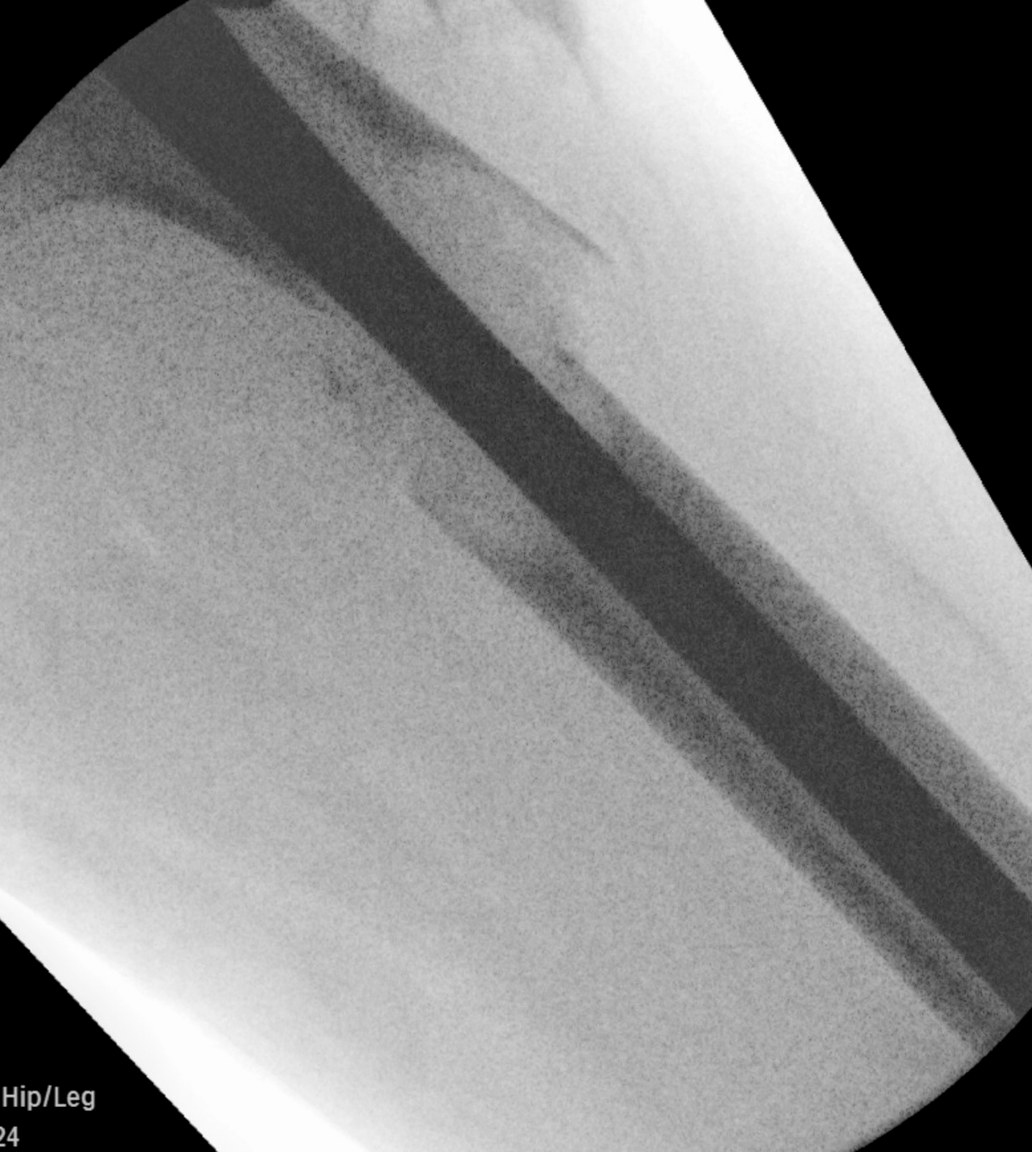
Intraoperative X-ray showing the long femoral gamma nail fixation of the left femoral shaft fracture

The surgery was initially planned for hospital day 3; therefore, enoxaparin was withheld on hospital day 2. However, the operation was delayed due to unforeseen emergencies in the operating theatre. After discussion with the hematology team, a decision was made to continue withholding enoxaparin on hospital day 3 and reschedule surgery for hospital day 4. This was a complex and high-stakes decision, as the balance between preventing thrombosis and managing bleeding risk was delicate, particularly in light of the active malignancy. On hospital day 4, the patient underwent successful open reduction and internal fixation (ORIF) with a long femoral gamma nail (Figure 4).

Postoperatively, the patient's hemoglobin dropped to 86 g/L on day 1 and further declined to 74 g/L on day 2, prompting transfusions. Over the following days, hemoglobin levels fluctuated between 74 and 79 g/L, despite multiple transfusions and close monitoring. The patient received a total of seven units of PRBC during the hospital stay, including two units administered preoperatively. The hemoglobin response remained suboptimal, and the patient developed wound-related concerns, including dressing soakage at the surgical site. In light of ongoing bleeding and the need to maintain hemoglobin stability, hematology recommended continuing enoxaparin at 40 mg twice daily rather than transitioning to the once-daily regimen initially planned.

Given persistently low hemoglobin despite transfusions and a transient episode of black stools, the patient underwent an extensive anemia workup, including gastroenterology input. A CT mesenteric angiogram and oesophagogastroduodenoscopy (OGD) on postoperative day 8 ruled out active gastrointestinal bleeding but revealed mild gastritis. By postoperative day 9, the hemoglobin level stabilized at 90 g/L. The anemia was later attributed partly to low folate levels (2.1 µg/L), and the patient was started on folate supplementation.

By postoperative day 12, wound oozing ceased completely, and hemoglobin levels continued to remain stable. The patient was transitioned to lifelong apixaban 2.5 mg twice daily as per hematology recommendations. She was discharged to an intermediary care setting for rehabilitation, with oncology follow-up arranged to continue assessment and complete further chemotherapy cycles.

## Discussion

Managing VTE in patients with cancer is complex, especially in the perioperative setting, where the balance between thrombotic and bleeding risks becomes even more critical. Thromboembolism in malignancy is multifactorial, involving procoagulant release by tumor cells, endothelial dysfunction, reduced mobility, and treatment-related effects [9]. Meanwhile, bleeding risk is heightened due to tumor invasion, thrombocytopenia, impaired liver function, and nutritional deficiencies. This case of metastatic urothelial carcinoma with pathological fracture and incidental DVT highlights the need for individualized anticoagulation strategies in such high-risk scenarios.

This case presented multiple layers of complexity: an incidental proximal DVT that altered the perioperative plan; fluctuating hemoglobin levels with ongoing transfusion needs; wound-related bleeding; extensive anemia workup revealing folate deficiency; multidisciplinary coordination; theatre delays due to system pressures; and the need for an individualized, evolving anticoagulation strategy. These challenges were further compounded by the lack of specific national or local guidance on perioperative anticoagulation dosing. Each of these hurdles required dynamic clinical judgment and close collaboration across specialties.

An incidental proximal DVT further complicated the perioperative decision-making. Because anticoagulation had to be paused around the time of surgery, the team discussed protective strategies, including the consideration of an IVC filter. While the routine use of IVC filters in cancer is controversial, they may be indicated in selected cases, particularly in high-risk surgical patients who cannot be safely anticoagulated [10,11].

Given the persistent anemia despite transfusions, the patient underwent an extensive workup, including imaging, endoscopy, and blood tests, which ultimately revealed significant folate deficiency. Common in elderly and cancer patients due to poor intake, malabsorption, or chronic loss [12], this deficiency was addressed with supplementation, which may not yield immediate hematologic improvement but is important for long-term recovery and overall nutritional support.

Multidisciplinary input was key to this patient's care. Hematology guided anticoagulation timing and dosage. Gastroenterology assisted in ruling out gastrointestinal bleeding. Orthopedic surgery coordinated operative decisions, while oncology contributed to long-term planning. This collaborative effort enabled flexible adjustments based on evolving risks, resulting in a safe clinical course.

Direct oral anticoagulants (DOACs) are now considered a suitable option for cancer-associated thrombosis in many patients, offering comparable efficacy to LMWH with the advantage of oral administration [3]. The enoxaparin dosing required repeated modification in response to perioperative bleeding risk, fluctuating hemoglobin levels, and wound-related concerns. Once the patient was clinically stable, apixaban was introduced for long-term secondary prevention. While LMWH has traditionally been preferred in malignancy, DOACs like apixaban are increasingly favored for selected patients due to ease of use and comparable outcomes, provided bleeding risk is carefully assessed [13]. The final anticoagulation strategy reflected a balance between bleeding risk, wound healing status, and the patient's overall prognosis.

This case emphasizes that managing cancer-associated thrombosis is not linear. It involves dynamic decision-making and careful coordination, especially in the perioperative setting. Guidelines provide a foundation, but deviations are often necessary due to patient-specific factors such as fluctuating clinical status, procedural delays, and unpredictable complications.

Ultimately, this case reinforces the importance of patient-centered care. It highlights that successful outcomes in complex oncologic patients depend not only on following protocols but also on timely reassessment, clear communication between specialties, and readiness to adapt. As more evidence emerges on DOACs and procedural strategies, individualized care remains the cornerstone of safe and effective thrombosis management in cancer.

## Conclusions

This case emphasizes the critical importance of individualized anticoagulation management in patients with DVT undergoing orthopedic procedures. The dynamic, real-time adjustments made throughout the patient's hospital stay were essential in preventing thromboembolic complications while managing bleeding risks. The multidisciplinary approach and close monitoring of the patient's clinical response were pivotal in achieving a successful outcome. This case reinforces the need for adaptive, personalized treatment strategies in managing complex oncologic and orthopedic patients.
